# Editorial: Nanomaterials based electrochemical sensors for hazardous pollutants detection

**DOI:** 10.3389/fchem.2022.1129787

**Published:** 2023-01-11

**Authors:** Dhammanand J. Shirale, Annamalai Senthil Kumar, Huimin Zhao

**Affiliations:** ^1^ Nanomaterials Processing Research Laboratory, Department of Electronics, School of Physical Sciences, Kavayitri Bahinabai Chaudhari North Maharashtra University, Jalgaon, India; ^2^ Nano and Bioelectrochemistry Research Laboratory, *CO*_2_ Research and Green Technology Centre, Department of Chemistry, School of Advanced Sciences, Vellore Institute of Technology, Vellore, India; ^3^ Key Laboratory of Industrial Ecology and Environmental Engineering, School of Environmental Science and Technology, Dalian University of Technology, Dalian, China

**Keywords:** electrochemical sensors, nanomaterials, chemically modified electrodes, hazardous pollutants, environmental pollution

**FIGURE 1:**
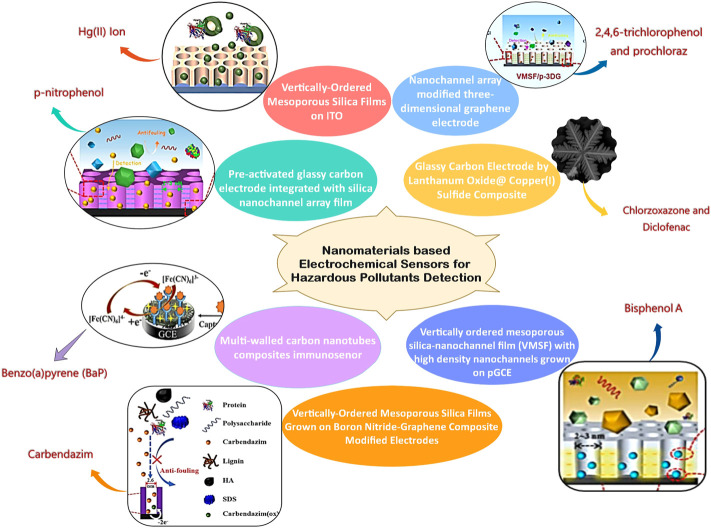
Cumulative representation of various sensors reported by the authors of this Research Topic.

Nanomaterials based electrochemical sensors for hazardous pollutants detection environmental pollution is a major global problem in the current era. It is essential to detect and analyze the hazardous pollutants present in the environment, which requires intensive research to develop fast, sensitive, selective, reproducible, and cost-effective technologies. Electrochemical sensors have received the utmost attention as this technology offers simplicity and cost-effective development with high sensitivity, selectivity, stability, reliability, and quick response. Taking into consideration the aforementioned qualities opens the way to field sensing. The electrochemical sensing mechanism seeks less potential/current, making the device portable for continuous on-site monitoring. From the perspective of environmental monitoring, there is a need for specific modifications of electrodes to enhance the features based on the pollutant present in the environment. The articles published in this Research Topic were reported about new electrochemical techniques used for the simultaneous detection of diclofenac and chlorzoxazone in drug tablets and urine specimens, direct and sensitive analyses of BPA in complex environmental samples, rapid and sensitive detection of carbendazim pesticides in real samples, immunosensor for detection of BaP in seawater and detection of Hg(II) ion in pharmaceuticals and soil samples. Figure 1 shows the cumulative representation of various sensors reported by authors of this Research Topic. Prochloraz has been extensively used in the production, storage, and transportation of various agricultural and forestry products for curing the plant diseases such as oil crops, cereals, tropical and subtropical fruits, vegetables, and various economic crops caused by pathogenic bacteria like Cercospora, Sclerotinia, Sclerotinia, Sclerotinia, *Fusarium* and Powdery mildew, Anthrax. It may cause skin irritation, severe disruption of mammalian endocrine levels, *etc.* Overexposure can also cause serious mutagenic, carcinogenic, or teratogenic effects on mammals. Zheng et al. demonstrate the ability of preactivated 3D graphene electrode nanochannel array for the effective detection of prochloraz and 2,4,6-trichlorophenol (TCP) found in the environment and food samples that adversely affect on human health. The developed VMSF/p-3DG sensor has got good sensitivity in the range 10 nM–15 *μ*M with 2.4 nM LOD. The simultaneous administration of various medications allows for the treatment of osteoarthritis and rheumatoid arthritis, as well as the reduction of painful symptoms associated with musculoskeletal problems. Single-pill combinations (SPCs) were developed for the simultaneous identification of coadministered medications in the presence of related substances and contaminants is emphasized in this Research Topic. The *La*_2_ *O*_3_ *@SF*−*LCu*_2_ *S* composites had much lower oxidation potential and higher oxidation currents. Baniahmad et al. have successfully designed chemically modified electrodes with a limit of detection (LOD) of 1.7 and 2.3 nM for the detection of diclofenac and chlorzoxazone, respectively.

One of the most commonly used chemical raw materials in the world is bisphenol A, also known as 2,2-bis(4-hydroxyphenyl) propane or BPA. It is frequently used as a monomer for the production of polymers (such as polycarbonate, polyphenylene ether resins, unsaturated polyester resins, *etc.*) or as an active component for the production of fine chemicals (such as plasticizers, flame retardants, antioxidants, heat stabilizers, rubber antioxidants, pesticides coating, *etc.* Constant exposure to BPA has been shown to adversely affect the neurological, immunological, and endocrine systems in humans and animals. It has also been shown to greatly raise the risk of numerous malignancies, including leukemia, prostate, and ovarian cancer. In light of the aforementioned Research Topic, Huang et al. have reported a vertically ordered mesoporous silica-nanochannel film (VMSF) with high-density nanochannels grown on the surface of an electrochemically activated glassy carbon electrode (p-GCE) by using the electrochemically assisted self-assembly (EASA) method for the electroanalytical application. Low detection limits enable the VMSF/p-GCE sensor to detect BPA at concentrations between 50 nM–1.0 *μ*M and 1.0 *μ*M–10.0 *μ*M. (LOD = 14.7 nM).

Carbendazim (CBZ), a low-cost insecticide with broad-spectrum action, is essential for managing weeds, illnesses, and pests in crop yields. It is challenging task to degrade CBZ, because of the benzimidazole ring’s stable properties. Due to the widespread and unchecked use of CBZ, CBZ residues have accumulated in the environment (such as soil and water), which might have long-term negative consequences on ecological safety and the aquatic ecosystem. Additionally, CBZ will have harmful effects on the body’s respiratory system and through direct contact, including skin inflammation, eye irritation, endocrine system disturbance, and hormonal imbalance. In a study by Zou et al. a nanocomposite of boron nitride-reduced graphene oxide (BN-rGO) as an adhesive and electroactive layer was used to demonstrate the steady growth of vertically ordered mesoporous silica films (VMSF) on the glassy carbon electrode (GCE). The oxygen-containing groups in rGO and the hybrid BN-2D rGO’s planar structure facilitate the steady development of VMSF *via* the electrochemically assisted self-assembly (EASA) technique.

The non-degradable nature of the compound, Mercury ion (*Hg* ^2+^), is the most hazardous metal in the ecosystem, making it a major threat to human health, and its contamination in food, medicine, and biological systems. *Hg* ^2+^ can enter the human body through a number of different pathways due to its high affinity for the Sulphur found in enzymes and proteins. Once inside the body, this metal can obstruct normal cell metabolism and cause damage to multiple organ systems, primarily the nervous and nephrotic systems, even at very low concentrations. Vertically ordered mesoporous silica films (VMSF) supported by the indium tin oxide (ITO) based chemically modified electrode surface were developed by electrochemically assisting self-assembly method and used for electrochemical detection of *Hg* ^2+^, according to a technique described by Zhang et al. Strong electrochemical signals are produced by VMSF due to its clear cationic selectivity and strong electrostatic interaction with *Hg* ^2+^, which are caused by the negatively charged channel walls and ultrasmall pore width. Using differential pulse voltammetry, anodic stripping voltammetric analysis of *Hg* ^2+^, was demonstrated with a quantitative detection range, (0.2 *μ*M–20 *μ*M) and a low limit of detection (3 nM).

Nitrophenol is a significant group of environmental contaminants and has a high degree of chemical stability, little capacity for poor biodegradation, and high toxicity. As an aromatic molecule, p-nitrophenol (p-NP) is frequently utilized as an intermediary in the production of fine chemicals such as insecticides, medications, and dyes. Based on the integration of vertically ordered mesoporous silica-nanochannel film (VMSF) over electrochemically pre-activated glassy carbon electrode, Su et al. developed an electrochemical sensing platform for the p-NP detection. Anodic oxidation at high voltage and cathodic reduction at low voltage are used in the green electrochemical polarization procedure to achieve the electrochemical pre-activation of GCE. The p-GCE has an expanded active region and newly added active sites with stable VMSF binding. With the electrochemically assisted self-assembly (EASA) approach, VMSF is produced quickly on p-GCE. With a limit of detection (LOD) of 9.4 nM, the newly designed VMSF/p-GCE sensor can sense p-NP in the ranges of 10 nM to 1 *μ*M and from 1 *μ*M to 30 *μ*M.

In marine oil spill contamination, benzo(a)pyrene (BaP) is the primary polycyclic aromatic hydrocarbon (PAH) that negatively affects both human health and marine ecosystems. The main cancer-causing PAH component, BaP, is composed of several benzene ring structures and has the ability to deposit in adipose tissue, the liver, and the kidneys. It typically needs metabolic activation to cause cancer. The bay region of BaP is vulnerable to enzymatic oxidation producing O-diol epoxides like BaP-2OH, which interact with proteins, DNA, and RNA and cause genotoxicity and carcinogenic activity. Yan et al. have fabricated a multi-walled carbon nanotube-chitosan composite loaded with a BaP antibody to create a new immunosensor. Based on a biosensing assay method, this immunosensor combines multi-walled carbon nanotubes chitosan composites as conductive mediators to improve the kinetics of electron transfer. The immunological response was then investigated by differential pulse voltammetry using the electrochemical signal generated by potassium ferricyanide as an electrochemical probe. Under ideal experimental circumstances, the peak current change had a detection limit of 0.27 ng mL and was inversely proportional to the BaP concentration in the range between 0.5 ng mL and 80 ng mL. These new electroanalytical systems provide a platform for electrochemical sensing of pollution in various other real samples.

